# Association of lymphocyte subsets with mortality in severe COVID‐19 pneumonia patients

**DOI:** 10.1002/jcla.24046

**Published:** 2021-10-09

**Authors:** Farzaneh Ashrafi, Pardis Nematollahi, Mehrzad Salmasi, Arash Hedayat, Babak Amra

**Affiliations:** ^1^ Hematology Oncology Division, Internal Medicine Department Isfahan University of Medical Sciences Isfahan Iran; ^2^ Department of Pathology, School of Medicine Isfahan University of Medical Sciences Isfahan Iran; ^3^ Internal Medicine Department Isfahan University of Medical Sciences Isfahan Iran; ^4^ Bamdad Respiratory and Sleep Research Center Department of Internal Medicine, Pulmonary and Sleep Ward Isfahan University of Medical Sciences Isfahan Iran

**Keywords:** COVID‐19, flow cytometry, T Lymphocytes

## Abstract

**Background:**

Few studies have investigated the alterations in the T and B cell counts and related subgroups in pulmonary infections especially COVID‐19. Here, we aimed to evaluate total T and B lymphocytes and T cell subgroup counts to find the possible correlation between number of these cells and severity and mortality in COVID‐19 patients.

**Methods:**

This study was performed on 40 patients with severe COVID‐19 infection confirmed by reverse transcription‐polymerase chain reaction (RT‐PCR) and chest HRCT in August 2020. By the time of admission, T lymphocytes profile in peripheral blood was investigated using multicolor flow cytometry. The total number of T lymphocytes, CD4+ T cells, CD8+ T cells, and B lymphocytes were calculated. Expression of CD2, CD3, CD5, and CD7 as pan T cell surface markers and expression of CD38 and HLA‐DR as activated markers on T lymphocytes were also evaluated.

**Results:**

Nine patients (22.5%) died during the study and 16 patients (40%) were admitted to ICU. Deceased patients demonstrated lower amounts of T cell count and CD4+ T cell count (with a marginal difference (*p* = 0.07)) compared with survived patients at the time of admission. The chance of mortality was significantly higher for patients with CD7 loss (OR = 14.89). A marginally significant relationship was also indicated between CD4<200/ml and mortality (OR = 8.65), but no other significant relationships were observed between variables and ICU admission.

**Conclusion:**

Altogether, CD7 loss on T lymphocytes and CD4+ T cell count below 200/ml revealed a significant relationship with mortality. Considering T lymphocytes and T cell subgroup count could have a predictive value for patients suffering from COVID‐19.

## Graphical abstract

CD7 loss on T lymphocytes and CD4+ T cell count less than 200/ml revealed a significant relationship with mortality in COVID‐19 patients. Considering T lymphocytes and T cell subgroups count could have a predictive value for patients suffering from COVID‐19.

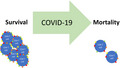

## 1 INTRODUCTION

Coronavirus is a large family of viruses that includes the common cold virus and the Severe Acute Respiratory Syndrome (SARS).^1^, ^2^ The novel coronavirus is a new respiratory coronavirus that emerged in late 2019 and early 2020 in Hubei province and Wuhan city of China and has worldwide prevalence.^3^ The virus is called “Severe Acute Respiratory Syndrome Coronavirus 2” (SARS‐CoV‐2). When the number of total deaths exceeded 1,000, the World Health Organization (WHO) declared the disease “Coronavirus disease 2019” (COVID‐19) on February 11, 2020.^4^ The SARS‐CoV‐2 virus is part of a large family of viruses (Coronaviruses) that are common in human beings and animals. The virus is transmitted from animals to human beings and, due to the behavior of the virus and its highly contagious nature, it quickly spreads through human‐to‐human contact all around the globe.^5^, ^6^

The role of T cell immune responses in disease pathogenesis and longer term protective immunity is currently poorly defined but is essential to understand to inform therapeutic interventions.^7^ The effectiveness of the immune response against viral infections depends on T cells' number and activity, which play an effective role in omitting the virus‐infected cells.^8^ Based on the evidence, CD8+ T lymphocytes play a critical role in eradicating acute pulmonary infectious diseases.^9^ Studies have also shown that leukopenia and lymphopenia could be observed in many patients with COVID‐19.^10^ Interestingly, it has been reported that within COVID‐19‐infected patients, CD4+ T cell and CD8+ T cell counts are significantly declined in more severe patients compared with moderate COVID‐19 infections.^11^

On the other hand, it has been stated that the proportion of the T cell response attributable to CD8+ (rather than CD4+) T cells is increased in mild infections, which is consistent with findings in another study showing a higher percentage of activated and proliferating CD8+ T cells in mild compared to severe COVID‐19.^12^, ^13^ Recent evidence also showed that a low percentage of circulating CD4+ and CD8+ T cell count might reflect the severity of the infection and is often accompanied by a poor prognosis. According to this study, peripheral blood CD4+ and CD8+ T cells' count could be a predictor biomarker for assessing disease and monitoring patients with COVID‐19.^14^ As a result, examining and evaluating the number of T cells and markers expressed on the surface of these cells can be a criterion for predicting disease progression.

Up to now, few studies have evaluated the available monitoring and prediction possibilities of CD4+ and CD8+ T cells in COVID‐19 infection. In this study, we aimed to evaluate total B and T cell count, T cell subgroups (CD4+ and CD8+ cells), expression of pan T cell markers, including CD2, CD3, CD5, CD7, and also some activated markers (CD38 and HLA‐DR) on T lymphocytes by multicolor flow cytometry, and examine its association with the disease severity and mortality in COVID‐19 patients. Thus we could set a possible criterion for predicting disease progression at the time of hospitalization; and also identify and anticipate the 1‐month outcome in patients who need more support and treatment.

## 2 METHODS AND MATERIAL

This study was performed in 2020 in Khorshid hospital, affiliated with Isfahan University of Medical Science, Isfahan, Iran. Hospitalized patients with severe COVID‐19 infection were included in the current study. The study protocol was approved and confirmed by the Research and Ethics Committee of Isfahan University of Medical Sciences (ethics code: IR.MUI.MED.REC.1399.276).

The inclusion criteria were age between 18 and 75 years, capillary oxygen saturation of 85% to 90% in room air, maximum saturation of 90% with a maximum of 6 L of nasal oxygen, oxygen saturation of 93–90% in room air or a respiration rate of more than 30 breaths per minute, and COVID‐19 positive test by reverse transcription‐polymerase chain reaction (RT‐PCR), and pathognomonic chest HRCT findings for COVID‐19 pneumonia. All patients had given the written informed consent to participate in this study. The exclusion criteria were intubation required within the first 24 h after admission, multiple organ failure, shock by the time of admission, immunodeficiency conditions, treatment with immunosuppressive drugs, and any underlying hematological disorder, and clinical and laboratory signs of infection other than COVID‐19.

A total number of 40 patients were included based on inclusion and exclusion criteria. Demographic data of patients, including age, gender, past medical histories such as previous chronic lung disease and smoking, were collected by the time of admission. Five milliliters of the patients' venous blood samples were collected in the Ethylenediaminetetraacetic acid (EDTA) Anticoagulated tubes before treatments.

Blood samples were analyzed using SIEMENS ADVIA 2120 for complete blood count (CBC). Cell viability was also evaluated by flow cytometry using propidium iodide staining. Samples with alive cells of more than 90% were accepted for immunophenotyping analysis. Multicolor flow cytometry device (PARTEK CYFLOW SPACE, Germany) and EXBIO antibody kits (Spain) were used for lymphocytes immunophenotyping. We used the following antibodies to evaluate cell surface markers: Anti‐CD2 (PE), Anti‐CD3 (PerCP, FITC), Anti‐CD4 (FITC, PE), Anti‐CD5 (FITC, PE), Anti‐CD7 (FITC), Anti‐CD20 (PerCP), Anti‐CD38 (FITC), Anti‐HLA‐DR (PE), Anti‐CD8 (PE, FITC). At least 10000 events were assessed for each sample. In FSC (Forward scatter) and SSC (Side scatter) plats, lymphoid cells were gated, and T lymphocytes, T helper cells, T cytotoxic cells, and B lymphocytes were separated and counted using CD3, CD4, CD8, and CD20 markers. Expression of CD2, CD3, CD4, CD5, CD7, CD8, CD38, and HLA‐DR markers was evaluated in T lymphocytes.

**FIGURE 1:**
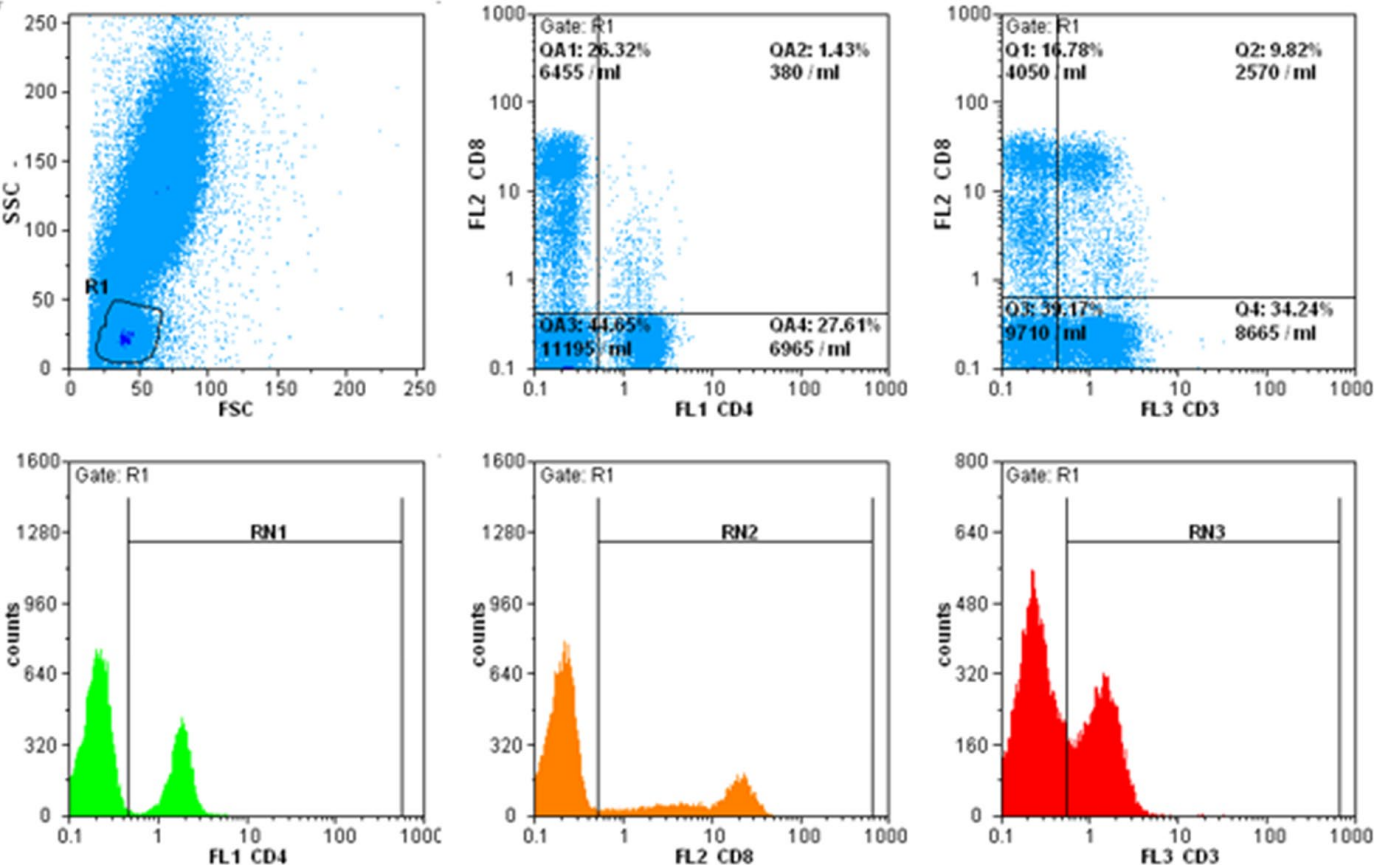
In the lymphocytic gate, CD3 positive T cells isolated in FL3 detector, CD4 positive cells in FL1, and CD8 positive cells in FL2 detectors

**FIGURE 2:**
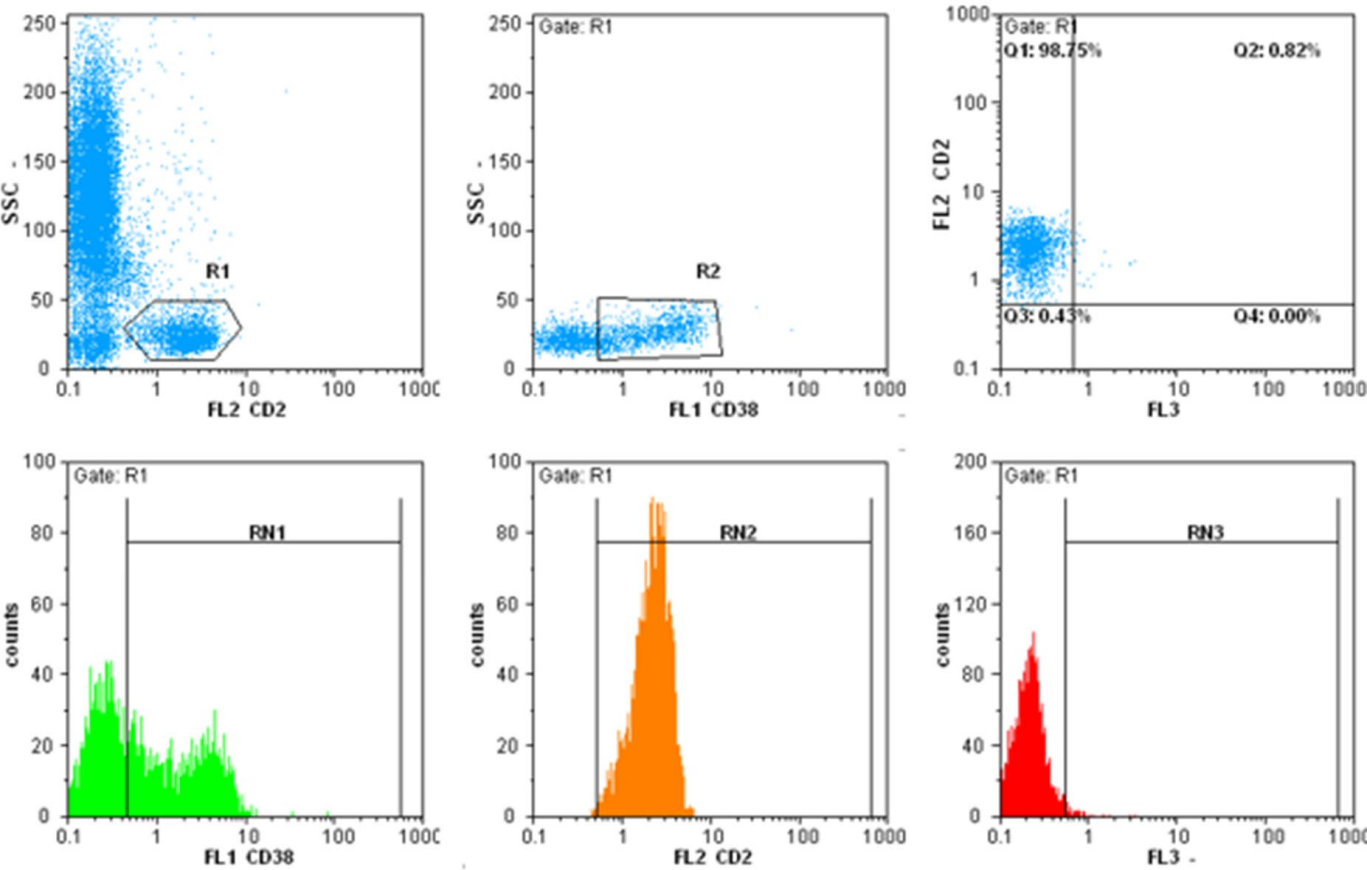
In the first dot plot, CD2 positive cells is separated (R1); in the second dot plot, CD38 expression on this population was evaluated (R2)

Given the fact that CD3 is a lineage‐specific T cell marker, the main marker for gating T cells was CD3. Cells were simultaneously stained with CD2, CD3, CD5, and CD7 to confirm the T cell gating and evaluate the loss of each surface marker. Cells that were positive for CD3 and at least one of CD2, CD5, and CD7 markers were considered as T cells. Representative flow cytometry plots are shown in Figures 1 and 2. The results of flow cytometry were analyzed using Flomax software based on the suggestion of the company. Expression of CD38 or HLA‐DR on more than 30% of T cells was regarded as activation and below 30% as nonactivation. We also considered marker loss when a marker was negative in more than 20% of the T cell population.^15^, ^16^

The treatment approach was based on the Iranian national treatment protocol for COVID‐19. Accordingly, patients received methylprednisolone 50 mg intravenously every 12 h for 3 days and then daily for 7 days, hydroxychloroquine 200 mg orally every 12 h for 5 days. For symptom control (pain, cough, nausea, and vomiting), acetaminophen codeine, diphenhydramine, dimenhydrinate, and promethazine were prescribed. All patients received enoxaparin 40 mg subcutaneously daily during hospitalization. The data according to disease progression, ICU admission, mechanical ventilation requirement, and mortalities were collected within 1 month. Patients discharged from the hospital were followed up with phone calls.

Our goal in this study was to evaluate the correlation between the counts of T cells and their subgroups and patients' outcome. Univariate and multiple logistic regression analyses were performed for mortality rate, ICU admission, and correlation between these outcomes and clinical and laboratory findings like T cell count and T cell CD markers expression. The significant level was at 5%.

Data were analyzed using Prism 7.05 (GraphPad). Patient groups were pairwise compared either to each other or to the reference group of healthy controls. Where data were normally distributed, an unpaired t‐test was used, and where that was not the case, the Mann–Whitney *U* test was applied. For categorical variables, Fisher's exact test was used. For analysis of longitudinal changes, two‐tailed paired t‐test was applied. The significant level was at 5%.

## 3 RESULTS

A total number of 40 patients entered the study based on inclusion and exclusion criteria. The primary analysis showed that the mean age of patients was 61.8 ± 10.5 years. Baseline demographic data, gender distribution, and initial clinical findings of patients are summarized in Table 1.

**TABLE 1:** Demographic and clinical data of patients

Variable		
Age (years) (mean ± SD)		61.8 ± 10.5
Time of starting symptoms (day) (mean ± SD)		6.8 ± 3.9
Time of ICU admission (day) (mean ± SD)		5 ± 2.7
Hospitalization duration (day) (mean ± SD)		13.5 ± 4.3
Gender (*N* (%))	Male	23 (57.5%)
	Female	17 (42.5%)
Vital signs	Systolic Blood Pressure	123.2 ± 17.4
	Diastolic Blood Pressure	74.7 ± 9.9
	Pulse Rate (PR)	87.6 ± 15.1
	Respiratory Rate (RR)	22.4 ± 3.7
	Temperature (T)	37.7 ± 0.8
	O2 saturation	87.4 ± 3.8
Mortality (*N* (%))	Yes	9 (22.5%)
	No	31 (77.5%)
ICU admission (*N* (%))	Yes	16 (40%)
	No	24 (60%)
Diabetes (*N* (%))	Yes	15 (37.5%)
	No	25 (62.5%)
Hypertension (*N* (%))	Yes	14 (35%)
	No	26 (65%)
Ischemic Heart Disease (IHD) (*N* (%))	Yes	5 (12.5%)
	No	35 (87.5%)
Smoker (*N* (%))	Yes	7 (17.5%)
	No	33 (82.5)
Pulmonary Disease (*N* (%))	Yes	2 (5%)
	No	38 (95%)

Comparison of data between patients with or without mortality and patients with or without ICU admission showed no significant differences between groups regarding age, time of starting symptoms, and vital signs (*p* > 0.05 for all items). However, there is a significant difference in gender distribution of patients admitted to ICU (the number of male patients was significantly higher) (*p* = 0.005). These data are demonstrated in Table 2.

**TABLE 2:** Comparison of demographic data and clinical characteristics among patients

	Mortality			*p* value	ICU admission		*p* value
	Yes		No		Yes	No	
Age	64.4 ± 11.5		62 ± 9.8	0.50	60.5 ± 13.4	63.4 ± 8.1	0.34
Time of starting symptoms	5.3 ± 2		7.5 ± 4	0.10	6.23 ± 2.9	7.5 ± 4.2	0.27
Systolic BP	116 ± 14		125.3 ± 17.1	0.11	120 ± 16.8	125.23 ± 17	0.30
Diastolic BP	75.6 ± 9.9		74.97 ± 9.0	0.84	75.64 ± 11.9	74.82 ± 7.6	0.76
PR	83.8 ± 14		89.46 ± 14.5	0.27	88.17 ± 14.9	88.44 ± 14.4	0.95
RR	22.5 ± 4.4		22.36 ± 3.7	0.92	22.58 ± 4.2	22.29 ± 3.7	0.80
T	37.99 ± 0.4		37.65 ± 0.9	0.30	37.86 ± 0.9	37.65 ± 0.9	0.43
O2 sat	80.8 ± 3.3		79.14 ± 3.8	0.21	79.63 ± 4.5	79.38 ± 3.4	0.81
CRP	76.55 ± 37.1		89.41 ± 56.6	0.52	95.55 ± 57.5	83.12 ± 52	0.45
Gender	Male	28.6%	71.4%	0.07	50%	50%	0.005
	Female	8.7%	91.3%		13%	87%	

Further evaluations indicated that patients with lower amounts of T cell count and CD4+ T cell count had higher mortality compared to other patients, and these differences were marginal. These data showed a mean T cell count of 335.9 ± 203.2 in dead patients and 539.7 ± 298.1 in survived patients and a mean CD4+ T cell count of 176.6 ± 98.1 vs. 331.1 ± 228.7 among dead and survived patients, respectively (*p* = 0.07 for both T cell and CD4). No other significant differences were observed between groups of patients regarding other indexes of flow cytometry (*p* > 0.05) (Table 3).

**TABLE 3:** Comparison of flow cytometry variables among groups

	Mortality		*p* ^1^	ICU‐admission		*p* ^1^	*p* ^2^
	Yes	No		Yes	No		
T cell count	335.9 ± 203.2	539.7 ± 298.1	0.07	401.5 ± 224.3	558.1 ± 315.4	0.10	0.07
B cell count	89.9 ± 74.7	123.4 ± 83	0.30	106.5 ± 81.3	122.8 ± 82.9	0.55	0.69
CD4 count	176.6 ± 98.1	331.1 ± 228.7	0.07	238.3 ± 144.8	337.7 ± 247	0.16	0.19
CD8 count	174 ± 171.1	178.7 ± 99.5	0.91	155.7 ± 132	191.5 ± 103.6	0.35	0.07
CD4/8 ratio	1.3 ± 0.6	1.9 ± 1.1	0.15	1.8 ± 1	1.8 ± 1.1	0.90	0.72
Aberrant CD38 On T cell	34 ± 12.6	27.7 ± 13.3	0.23	29 ± 14.8	29.1 ± 12.4	0.98	0.35
Aberrant HLA‐DR On T cell	18.6 ± 8	13.8 ± 12.2	0.30	14 ± 7.9	15.3 ± 13.5	0.73	0.63

Note

*p* ^2^ = Adjusted for gender.

As mentioned earlier, the expression of CD38 or HLA‐DR more than 30% was regarded as activation and below30% as nonactivation. Additionally, marker loss was defined as a reduction in each of CD3, CD5, and CD7 markers in more than 20% of T cells. Based on our results, higher risks of mortality were observed among patients with aberrant expression of CD38 lower than 30% or patients with CD7 loss or CD4+ T cell<200/μl. Multiple analysis on CD4+ T cell count, CD38 aberrant expression, and CD7 loss and adjusting them based on gender showed a significant relationship between CD7 loss with mortality (OR(%95 CI) = 14.98(1.09–205.46), *p*‐Value = 0.02). A marginal relationship was also indicated between CD4+ T cell<200/μl and mortality (OR(%95 CI) = 8.65 (1.80–93.35), *p*‐Value = 0.03), but no other significant relationships were observed between the mentioned variables and ICU admission (Table 4).

**TABLE 4:** Assessments of relationships between flow cytometry variables with ICU admission and mortality

		ICU admission	Univariate OR (95% CI)	Multiple OR (95% CI)	*p*‐value	Mortality	Univariate OR (95% CI)	Multiple OR (95% CI)	*p*‐value
CD4/CD8 ratio	>2	6 (40%)	1.067 (0.47–2.39)	–	–	2 (13.3%)	0.533 (0.123–2.30)	–	–
	<2	9 (37.5%)				6 (25%)			
CD4 count	>200	7 (30.4%)	2.28 (0.6–8.57)	–	–	2 (8.7%)	6.3 (1.07–36.93)	8.65 (1.80–93.35)	0.03
	<200	8 (50%)				6 (37.5%)			
Aberrant CD38	pos	8 (53.3%)	1.829 (0.839–4)	–	–	6 (40%)	4.8 (1.11–20.762)	5.19 (1.66–40.56)	0.049
	neg	7 (29.2%)				2 (8.3%)			
Aberrant HLA‐DR	pos	2 (40%)	1.015 (0.32–3.219)	–	–	2 (40%)	2.2 (0.603–8.03)	–	–
	neg	13 (39.4%)				6 (18.2%)			
CD3 loss	Yes	0 (0%)	NC	–	–	0 (0%)	NC	–	–
	No	15 (45.5%)				8 (24.2%)			
CD5 loss	Yes	1 (50%)	0.757 (0.178–3.214)	–	–	1 (50%)	0.378 (0.081–1.762)	–	–
	No	14 (37.8%)				7 (18.9%)			
CD7 loss	Yes	3 (60%)	2.75 (0.4–18.8)	–	–	3 (60%)	8.7 (1.15–65.93)	14.98 (1.09–205.46)	0.02
	No	12 (35.3%)				5 (14.7%)			

Abbreviation: NC, Not computable.

## 4 DISCUSSION

COVID‐19 infection involves multiple organs and affects different systems, including the immune system. It has been well established that this disease causes significant lymphocyte changes, especially a decline in T cells. During COVID‐19 infection, the immune system is highly activated, leading to massive production of pro‐inflammatory cytokines such as interleukin (IL)‐1β, IL‐6, and tumor‐necrosis factor (TNF)‐α and activation of T lymphocytes. Dysregulated immune response and immune cells have been reported in COVID‐19 and also other conditions such as chronic hepatitis C virus (HCV)‐infected patients receiving direct‐acting antivirals^17^ and herpes simplex virus‐1 (HSV‐1) infection,^18^ chronic viral infections,^19^ and several human acute respiratory viral infections.^20^ Various studies have been performed on prognostic values of these changes in severe infections such as COVID‐19. By using the prognostic factors in infected patients, we would be able to identify patients who may require ICU admission and provide more supportive care and effective treatments.

Here, in this study, we investigated the correlation of T cell and subgroups count with patients' outcomes in 40 cases of COVID‐19 pneumonia. We indicated that the number of male patients admitted to ICU was significantly higher than females. Further evaluations also indicated that patients with lower total T cell and CD4+ T cell counts had higher mortality rates (*p* = 0.07 for both). Based on our results, higher mortality risks were observed among patients without CD38 aberrancy (lower than 30%) or patients with CD7 loss or CD4 below 200/μl. The importance of lymphocyte subset counts and their relation to disease severity has been addressed in previous studies. Lymphocyte subset alteration was associated with clinical characteristics and treatment efficacy of COVID‐19. Most studies have declared that lymphopenia and increased levels of certain cytokines, such as IL‐6, have been closely associated with the disease severity.

Based on our data, patients with mortality due to severe COVID‐19 infection had significantly decreased amounts of T cell count and CD4+ T cell count compared to others. A novel meta‐analysis by Huang and colleagues showed that absolute counts of major lymphocyte subsets are significantly and substantially decreased in severe COVID‐19 disease. It was also explained that CD4+ T cell, CD8+ T cell, B cell, NK cell, and total lymphocyte cell counts all showed a statistically significant reduction in patients with severe/critical COVID‐19 disease compared to mild/moderate disease.^21^ Our findings are somehow in line with these results. However, the mentioned meta‐analysis evaluated the association of lymphocyte count with severity in COVID‐19 patients, while our study found an association between lymphocyte count and mortality in COVID‐19 patients. Besides, the novelty of our study, which is not evaluated in the majority of related studies, is investigating the loss of T cell surface markers in COVID‐19 patients. Loss or aberrant expression of T cell markers is reported in some viral diseases such as infection with human T‐cell leukemia virus type‐1 (HTLV‐1) and Epstein Barr virus (EBV),^22^, ^23^, ^24^ and also hematologic malignancies.^25^, ^26^, ^27^ Several reports have associated the severe COVID‐19 with hemophagocytic lymphohistiocytosis (HLH).^10^, ^28^, ^29^ Given that the loss of T cell surface markers is also reported in the HLH,^24^ our findings might suggest a further association between severe COVID‐19 infection and HLH. However, future investigations are required to investigate the association of severe COVID‐19 with HLH.

Wang and others also evaluated and compared different immune cell markers in COVID‐19 patients and showed that total lymphocytes and CD4+ T cells significantly decreased in COVID‐19 patients, and severe cases had a lower level than mild cases. They also showed that the CD4/8 ratio was not found to be significantly different between the two groups.^30^ These findings are also somehow in line with the results of our study.

Some other studies have reported different results compared to our data. A study by Chan and others declared that COVID‐19 patients, in general, had significantly lower total lymphocytes, and CD4+cells and CD4/8ratio were not significantly different among patients. They also stated that the levels of CD8+ cells, B cells, and NK cells were significantly lower in these patients.^31^ Wan and others also showed that the levels of CD4+ and CD8+ T cells significantly decrease in severe COVID‐19 infection compared to mild patients. It was also shown that no significant differences were observed between patients regarding B cells, NK cells, and CD4/8 ratio.^32^

Another study by Zheng and colleagues evaluated epidemiological characteristics and clinical features of 32 critical and 67 noncritical cases of COVID‐19 and showed that critically ill patients have significantly lower white blood cells, neutrophils, and lymphocytes; and lower CD4 and CD8 counts. It was suggested that such markers could be used for evaluating the prognosis of patients.^33^

To date, the main reason for lymphopenia and changes in lymphocyte markers in COVID‐19 infections remains unknown. It has been expressed that the decrease of lymphocytes is a result of activation‐induced apoptosis or aggressive migration from peripheral blood to the lungs, where robust viral replication occurs.^34^ The main etiology of this condition is thought to be due to the trafficking of activated lymphocytes into inflamed tissues,^35^ but further studies are still required.

In this study, no significant alterations were observed in the T cell count and immune markers in patients admitted to ICU, but significant changes were observed in patients with mortality. These results are in line with most of the previous findings suggesting the importance and prognostic use of T cell and CD4+ cell count in patients with COVID‐19. The differences between our results and similar studies could be mostly due to variations in study populations.

We observed that the death cases progressed rapidly to acute respiratory distress syndrome, septic shock, difficult‐to‐correct metabolic acidosis, coagulopathy, and multiple organ failure. In general, the severity of disease for most patients could be determined according to these clinical characteristics, but laboratory data and immune marker alterations could play pivotal roles in determining the prognosis of patients. As we showed, significant relationships were observed between CD7 loss and CD4+ T cell count below 200/μl with mortality. Significant reductions in total lymphocytes and CD4+ T cells were reported by Gallo Marin and colleagues in a literature review. It was also showed that CD7 loss and CD4+ T cells <200/μl could increase the chances of disease severity and mortality.^36^ Our data were in line with these findings. To date, very little attention has been given to the prognostic roles of CD7 loss in COVID‐19. Parasole and others declared that CD7 loss could be indicated in patients with severe infections.^37^

According to this study, CD38 aberrant expression (higher than 30%) is associated with higher mortality risk. Tang and colleagues and Miller and colleagues showed a significant reduction in CD38 levels in patients with more severe COVID‐19 infections and mortalities.^38^, ^39^ Based on these studies, CD38 could be used to monitor high‐risk patients, but other markers and lymphocyte counts should also be determined. Another finding of this study was that the number of male patients admitted to ICU was significantly higher than females. In a recent study by Conti and others, it was discussed that women are less vulnerable to COVID‐19 infection due to differences in the immune system and immune receptors. Based on the evidence, sexual differences can affect several aspects, such as antiviral immune response, morbidity, transmission, and pathogenesis. Estrogen treatment prevents osteoporosis by inhibiting the cytokine pathway necessary for the differentiation and activation of osteoclasts. It was also stated that Estrogens modulate receptor response and pro‐inflammatory cytokine production.^40^ This issue is known to be caused by the immune regulatory genes encoded by X chromosome in females. As a result, lower viral load levels and less inflammation are observed in women compared to man, while CD4+ T cells are higher with better immune response. Sharma and colleagues also described that sex differences could affect the outcomes of patients, but other comorbidities, viral load, and severity of the infection are more important factors.^41^

One of the limitations of this study was that the current project was conducted in Isfahan city, while many other cities in Iran are involved with COVID‐19. We also had a local study population compared to other studies.

## 5 CONCLUSION

We suggest that T cell count, CD4+ T cell count, CD7 loss, and aberrant CD38 on T cells could play a prognostic role for mortality assessments in COVID‐19 patients. However, more studies on larger populations seem necessary to confirm these findings. We also suggest that more attention should be given to patients in critical condition due to low CD4+ T cell counts and increased risk of opportunistic infections and lower antiviral immune surveillance. T cells' and subgroups' immune profiles are laboratory variables that may be valuable prognostic test in survival and outcome of severe COVID‐19 pneumonia infection.

## CONFLICT OF INTEREST

The authors declare no conflict of interest.

## AUTHOR CONTRIBUTIONS

F.A. conceived and supervised the research and its design. A.H., P.S., and M.S. designed and performed the experiments, interpreted the data, and wrote the manuscript. A.H., M.S., and B.A. assigned reagents/materials/analytical tools. All authors approved the final manuscript.

## Data Availability

The data that support the findings of this study are available from the corresponding author upon reasonable request.
